# Within- and between-session reliability of pelvic marker placement and posture in lower-limb amputees

**DOI:** 10.33137/cpoj.v8i2.46063

**Published:** 2025-10-20

**Authors:** A Withey, D Cazzola, A Tabor, E Seminati

**Affiliations:** 1 Department for Health, University of Bath, Bath, UK.; 2 Faculty of Health and Applied Sciences, University of the West of England, UK.

**Keywords:** Lower-Limb Amputation, Gait Analysis, Posture, Kinematics, Longitudinal Consistency, Marker Placement Reliability

## Abstract

**BACKGROUND:**

Accurate placement of anatomical markers is essential for valid three-dimensional (3D) gait analysis, yet individuals with lower-limb amputation (LLA) pose unique challenges due to altered anatomy, prosthetic interfaces, and increased adiposity.

**OBJECTIVE:**

This study assessed within- and between-session reliability of pelvis marker placement and static posture kinematics in adults with unilateral LLA.

**METHODOLOGY:**

Fourteen adults with unilateral LLA (age: 58 ± 15 years, height: 174.6 ± 7.5 cm, body mass: 91.1 ± 27.7 kg, BMI: 29.6 ± 7.5 kg/m^2^; eleven transtibial, three transfemoral) participated in two sessions spaced 3–13 months apart. Reliability of marker distances and static posture kinematics were assessed using intraclass correlation coefficients (ICC) and standard error of measurement (SEM).

**FINDINGS:**

Within-session reliability of pelvis marker distances was good to excellent (ICC ≥ 0.78), whereas between-session reliability was lower (ICC as low as 0.14), particularly for posterior superior iliac spine markers. Pelvis kinematics demonstrated moderate reliability within sessions (average ICC ≈ 0.71), but trunk kinematics showed poor reliability. SEM values were low (<5°), suggesting acceptable absolute consistency despite variable ICCs, likely driven by postural changes and prosthetic factors.

**CONCLUSION:**

Findings support reliable pelvis marker placement within sessions but highlight challenges for longitudinal consistency. Multiple trial collections and standardised posture protocols are recommended to improve long-term reliability.

## INTRODUCTION

Correct placement of anatomical markers in three-dimensional (3D) gait analysis is crucial for ensuring valid biomechanical outcomes. However, within- and between-sessions variability in markers placement can introduce errors in kinematic movement analysis, especially in longitudinal studies where participants are monitored repetitive times across different sessions, such as in the monitoring of patients' rehabilitation.^1^ Research has shown that human error in inconsistent markers placement can affect kinematic gait data in terms of average joint angles by up to 75%.^1^

Together with marker placement, a correct static trial is a fundamental step in motion analysis, as it establishes segment lengths at the start of a measurement, which are essential for many kinematic and kinetic gait calculations.^2,3^ It also provides a reference for the participant's anthropometrics and anatomical alignment, which helps define body segment coordinate systems with respect to the ground, and it allows comparison of data across participants or trials by providing a standardized reference. Therefore, exploring the reliability of within- and between-session anatomical marker placement and static posture with respect to kinematic outcomes is an important area of study when designing longitudinal studies.

While marker placement reliability has been well-documented in the general population with skin marker placement variability (standard deviation) within 10 mm and 12 mm for intra-evaluator and inter-evaluator, respectively,^4^ limited research has examined its consistency in individuals with lower-limb amputation (LLA). This population presents unique challenges due to altered anatomy, soft tissue distribution, prosthetic limb interfaces and suspensions (especially for above knee amputations), and limb differences, all of which may influence marker placement precision and subsequent biomechanical interpretations. Moreover, a substantial proportion of individuals with LLA are overweight or obese, with prevalence rates ranging from 28% to 48%.^5,6^ Excess subcutaneous fat can obscure anatomical landmarks, causing inconsistent marker placement and errors in joint kinematics.^7,8^

Despite these known challenges, the reliability of pelvis marker placement among individuals with LLA remains largely unexplored, particularly in those with higher BMI.

This study focused primarily on evaluating the test-retest reliability of pelvis marker placements within- and between-sessions in individuals with LLA, together with static trial posture variability for the pelvis and trunk segments. We expected pelvis marker placement to demonstrate reliability, with ICC values exceeding 0.70 across a diverse sample of participants with LLA and varying body mass index (BMIs). An intraclass correlation coefficient (ICC) value of 0.70 or higher is acceptable for internal consistency and reliability in this population.^9-11^

## METHODOLOGY

Thirteen males and one female with unilateral LLA (age: 58 ± 15 years; height: 174.6 ± 7.5 cm; weight: 91.1 ± 27.7 kg; BMI: 29.6 ± 7.5 kg/m^2^) were recruited; eleven transtibial and three transfemoral (**Table 1**). Participants were classified per National Health Service (NHS) Body Mass Index (BMI) guidelines: seven as obese (BMI ≥ 30), two as overweight (BMI 25–29.9), and five as healthy weight (BMI < 25). Ethical approval was obtained (REC 23/EE/0090).

**Table 1:** Individual participants' characteristics at session 1 and session 2. Mean values ± standard deviation (SD) are reported at the end of the table.

Participant	Limb-loss level	Cause of limb loss	Sex	Height (cm)	Age (years)		Mass (kg)		Time since limb loss (weeks)	
					Session 1	Session 2	Session 1	Session 2	Session 1	Session 2
P01	TT	Traumatic	M	168	39	40	87	73	18.7	70.8
P02	TT	Vascular	M	168	84	85	59	62	16.1	58.1
P03	TF	Sepsis	M	160	70	70	59	62	19.4	32.0
P04	TT	Vascular	M	173	75	76	91	90	34.7	87.6
P05	TT	Vascular	M	179	48	49	111	106	27.3	53.3
P06	TT	Vascular	M	185	43	44	97	105	14.1	66.8
P07	TF	Vascular	M	179	53	54	68	78	11.4	50.3
P08	TT	Cancer	M	178	46	47	110	94	14.1	52.7
P09	TT	Chronic pain	M	173	42	42	108	107	19.9	59.8
P10	TT	Vascular	M	183	46	46	162	134	31.4	43.8
P11	TT	Vascular	M	185	69	69	105	101	19.3	59.2
P12	TT	Vascular	M	175	72	73	69	74	18.0	70.1
P13	TT	Vascular	M	173	67	68	69	73	13.4	59.4
P14	TF	Vascular	F	165	54	55	80	83	15.1	67.0
**Average**				**175 ± 8**	**58 ± 15**	**58 ± 15**	**91 ± 28**	**89 ± 20**	**19 ± 7**	**59 ± 13**

Participants were recruited from the NHS Bristol Centre for Enablement and the Portsmouth Enablement Centre. Recruitment commenced in July 2023 and continued for 12-months. Clinicians who were part of the participants' routine care team identified eligible patients during assessment days according to the inclusion and exclusion criteria.

### Inclusion and exclusion criteria

Participants were included if they were over 18 years of age, had a unilateral lower-limb amputation (above or below the knee), were newly fitted with a prosthesis (three months post-amputation) and were able to walk on level ground. Exclusion criteria were balance disorders, congenital lower-limb absence, inability to provide consent for prosthetic use and individuals at serious risk of complications to the sound limb that could limit normal rehabilitation. These clinicians made the initial approach to potential participants and introduced the study. The primary investigator of this study (AW) then provided information sheets and informed consent forms to eligible participants.

### Experimental set-up

A motion capture system (Qualisys, Sweden) with eight infrared cameras was used to detect retroreflective markers. The system was calibrated prior to data collection using a Qualisys calibration wand and L-frame, achieving a residual error of < 1 mm (0.70 ± 0.10 mm). Cameras recorded at a sampling rate of 200 Hz, and the global coordinate system was aligned with a fixed floor platform (600 x 400 x 35 mm). For each static trial described in session 1 and 2, the participants were asked to stand for 10 seconds in the same anatomical reference position relative to the platform.

### Protocol

**Figure 1:**
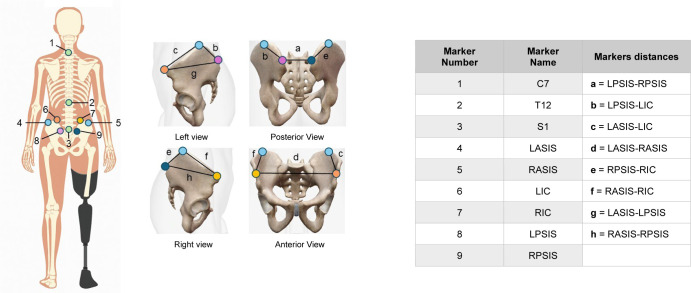
Marker placement positions is represented from the posterior view in the full body diagram on the left. Marker details and 3D Euclidean distances are listed and represented in the right side of the picture.

Participants completed two motion capture sessions (≈ 45 minutes each). In both sessions, a single evaluator with three years of experience in collecting 3D motion capture data and running sessions on individuals with LLA, applied nine pearl retroreflective markers (diameter: 15.9 mm) to the pelvis and trunk: bilaterally on the anterior superior iliac spine (ASIS), posterior superior iliac spine (PSIS), and iliac crest (IC), plus spinous processes at S1, T12, and C7 (**Figure 1**). Markers were affixed directly to the participants' skin with double-sided adhesive tape to avoid movement relative to the underlying anatomical landmarks during motion capture. For the three transfemoral amputees, the pelvis markers were instead placed on the prosthetic suspension belt (TES Suspension Belt, Ortho Europe, UK). Each participant completed repeated sessions at approximately the same time of day to minimize within-subject variability, however, session times were not standardized across participants due to the use of two clinics and room availability.

- **Session 1:** 3–6 months post-amputation (3.9 ± 1.1 months): One 10-second static standing trial (*Static 1*) was recorded. At this time, all participants had commenced rehabilitation and were able to ambulate safely with their prosthesis.
- **Session 2:** ≥ 3 months later; range 7–16 months (13.1 ± 2.7 months). Three 10-second static standing trials were recorded:
  - *Static 2:* Initial standing trial with full marker set.
  - *Static 3:* After removing/reapplying all markers except ICs (after 10-minute rest).
  - *Static 4:* After 12 walking gait trials (≈ 30 minutes after static 2), markers unchanged.

IC markers were retained in Session 2 to provide a consistent reference point for calculating placement error when the remaining pelvic markers were reapplied.

### Data analysis

Markers were labelled in Qualisys Track Manager (Qualisys, Sweden). OpenSim 4.5 was then used to create a skeletal model of the pelvis and trunk segments, which was scaled to each participant's standing calibration trials for each session. The pelvis segment was defined by the bilateral ASIS and PSIS markers, with the IC markers used as additional references as mentioned previously. The trunk segment was defined using the spinous process markers at S1, T12 and C7. The 3D Euclidean distances were calculated between pelvis markers, including the distances between the ASIS and PSIS markers and the fixed iliac crest markers (**Figure 1**). Additionally, pelvis (relative to the global reference system) and trunk (relative to the pelvis segment) joint angles were calculated for the standing calibration trials.

### Statistical analysis

The reliability of marker placement and pelvis and trunk kinematic parameters was assessed using the ICC from a two-way random model (ICC_2, k_) and standard error of measurement (SEM). ICC thresholds were considered: poor (< 0.50), moderate (0.50–0.75), good (0.75–0.90), and excellent (> 0.90).^12^ For this study, an ICC value ≥ 0.70 was deemed acceptable.^9-11^ This threshold has also been applied in previous research examining marker placement during gait analysis, a methodology closely related to the present study.^10^ Standard Error of Measurement (SEM) was calculated as the square root of the mean square error term from a repeated measures ANOVA.^13^ Pelvis and trunk angle errors (%) were calculated as the absolute difference in range of motion (ROM) between repeated measurements, expressed as a percentage of the ROM of the first measurement. This method was used for both within-session comparisons (repeated trials within the same session) and between-session comparisons (measurements from different sessions). All analyses were performed in MATLAB^®^ R2021b (MathWorks, Inc., USA).

## RESULTS

### Within-session results

Within-session ICCs for pelvis marker distances (session 2, statics 2–4) ranged from 0.78–1.00, indicating good to excellent reliability, with the highest reliability coefficients measured for LASIS-RASIS and LASIS-LPSIS distances (**Table 2; Appendix 1**). ASIS–IC and PSIS–IC distances had SEMs < 4 mm (**Table 3**). Pelvis angles had ICCs averaging 0.71 ± 0.19, with tilt and list both ≥ 0.70 (**Appendix 2**) and SEMs < 5°. Static trials 3 and 4 differed by < 0.5° for all pelvis angles. For trunk range of motion parameters, all parameters displayed SEM values below 5°, however trunk rotation showed the lowest ICC values (**Table 2; Appendix 3**).

**Table 2:** Reliability indices (ICC) of ASIS and PSIS marker placement Euclidean distances, and pelvis and trunk kinematic parameters. Values represent the range of ICCs observed within- and between-sessions across static trials.

Measure	Within-session ICC	Between-session ICC
LASIS-LIC	0.78–0.81	-
RASIS-RIC	0.78–0.97	-
LPSIS-LIC	0.90–0.98	-
RPSIS-RIC	0.82–0.91	-
LASIS-RASIS	0.94–1.00	**0.68**–0.76
LPSIS-RPSIS	0.80–0.97	**0.14–0.34**
LASIS-LPSIS	0.95–1.00	**0.68**–0.71
RASIS-RPSIS	0.97–0.98	**0.59–0.67**
Pelvis tilt	0.79–0.95	**0.23–0.23**
Pelvis list	0.81–0.91	**-0.04–0.01**
Pelvis rotation	**0.43–0.70**	**-0.17–0.41**
Trunk flexion/extension	0.75–0.85	**-0.01–0.17**
Trunk lateral bending	0.74–0.95	**0.15–0.32**
Trunk rotation	**0.49**–0.84	**-0.13–0.29**

ICC: Intraclass correlation coefficient; ICC values of ≤ 0.70 are presented in bold.

**Table 3:** Euclidean distances reported in mm as mean ± standard deviation and Standard Error of Measurement (SEM) calculated between and within sessions.

Distance	Static 1	Static 2	Static 3	Static 4	SEM (within)	SEM (between)
LASIS-LIC	-	107.2 ± 26.0	118.0 ± 29.9	118.0 ± 29.4	2.4	-
RASIS-RIC	-	104.0 ± 26.3	102.3 ± 24.1	103.6 ± 25.7	2.6	-
LPSIS-LIC	-	195.1 ± 22.8	194.0 ± 24.2	194.2 ± 26.4	1.7	-
RPSIS-RIC	-	209.5 ± 26.3	209.4 ± 19.9	213.0 ± 20.3	2.0	-
LASIS-RASIS	326.8 ± 59.7	326.9 ± 42.3	326.9 ± 47.0	327.9 ± 46.3	2.3	5.5
LPSIS-RPSIS	81.6 ± 17.9	85.7 ± 15.1	88.5 ± 11.7	88.9 ± 11.1	1.3	2.7
LASIS-LPSIS	257.7 ± 34.6	252.7 ± 29.4	256.2 ± 33.1	256.7 ± 32.9	1.3	3.6
RASIS-RPSIS	259.5 ± 32.1	260.4 ± 28.6	259.0 ± 29.0	262.4 ± 28.5	1.2	3.6

### Between-session results

Between-session reliability (session 1 vs. 2) showed lower ICCs (**Table 2; Appendix 4**), ranging from 0.14–0.76, though SEMs remained acceptable. The lowest ICC value (0.14) was for LPSIS–RPSIS distance. Left and Right ASIS distance had the highest SEM (5.5 mm), suggesting more variation across timepoints (**Table 3**), although kinematic measurements consistently reported very small SEMs. Pelvis and trunk parameters had low ICCs (**Appendix 5** and **Appendix 6**), but SEMs remained <5° (**Table 4**).

**Table 4:** Pelvis and trunk kinematics for the pelvis and the trunk segments, in degrees as mean ± standard deviation and Standard Error of Measurement (SEM) calculated between and within sessions.

Pelvis/trunk kinematics	Static 1	Static 2	Static 3	Static 4	SEM (within)	SEM (between)
Mean pelvis tilt	7.7 ± 13.1	-0.7 ± 6.3	0.6 ± 4.9	0.2 ± 5.9	0.54	1.50
Mean pelvis list	1.1 ± 5.1	2.9 ± 3.4	2.3 ± 3.3	2.6 ± 3.4	0.31	0.84
Mean pelvis rotation	3.1 ± 4.0	1.6 ± 3.0	0.1 ± 3.9	0.1 ± 3.5	0.57	0.75
Mean trunk flexion	-0.3 ± 0.3	-0.2 ± 0.1	-0.2 ± 0.1	-0.2 ± 0.1	0.01	0.04
Mean trunk list	-0.0 ± 0.1	-0.0 ± 0.1	-0.0 ± 0.1	-0.0 ± 0.1	0.01	0.01
Mean trunk rotation	0.1 ± 0.2	0.0 ± 0.1	0.0 ± 0.1	0.0 ± 0.1	0.01	0.03

## DISCUSSION

This study assessed the within- and between-assessor reliability of pelvis marker placement and kinematics in individuals with LLA, a population with anatomical and practical challenges limiting accurate and consistent marker positioning.

Within-session pelvis marker distances showed good to excellent reliability (ICCs ≥ 0.78), supporting use in a single-day or repeated trials. Between-session reliability was lower (ICCs as low as 0.14), especially for PSIS markers, likely due to anatomical variation, prosthetic interfaces, and increased adiposity obscuring landmarks. In this context, “prosthetic interfaces” refers to the components of the prosthetic socket and suspension system that interface with the residual limb. These interfaces can alter the positioning or soft tissue conformation around the pelvis between sessions, especially for transfemoral amputees. Despite this, SEMs remained low (< 4 mm), indicating limited absolute error.

The time gap between testing sessions 1 and 2 (ranging from three to six months post-amputation for the initial session to seven months or more for the follow-up) may have contributed to the observed inconsistencies. In addition, changes in static standing posture due to the prosthetic limb, body mass fluctuations (change of up to 2.4 kg between sessions in the current study), prosthetic discomfort, and fatigue, along with external factors like suspension systems or high BMI, may have affected consistent marker placement and segment orientation across sessions on a population level. These factors can obscure anatomical landmarks and cause marker displacement — especially over longer intervals between assessments — reducing the reliability of pelvis and trunk kinematics. This likely contributed to the high placement variability observed in our study (standard deviations: 11.1–59.7 mm), which exceeds the 10–12 mm values reported in healthy individuals, where variability is primarily attributed to soft tissue artifact.^4^ In our case, marker placement also affected joint angles calculation during dynamic trials. Between-session pelvis errors were 18% (tilt), 20% (list), and 10% (rotation), versus within-session errors of 10%, 7%, and 2%. Trunk angles showed even greater variability, with between-session errors of 22%, 34%, and 61%, and within-session errors of 6%, 18%, and 37%. Subtracting a standing baseline from pelvis and trunk kinematics could reduce the apparent variability in walking trials by accounting for inter-session or inter-trial differences in participants' neutral posture. Because trunk angles were calculated relative to the pelvis, any variation in pelvic orientation during standing contributes directly to trunk angle variability. By referencing walking kinematics to a consistent static posture, between- and within-session errors in both pelvis and trunk angles might be reduced, particularly for rotational measures, as individual differences in habitual standing alignment would be removed. However, this approach would not eliminate variability due to marker placement, soft tissue artifact, or dynamic postural adjustments during gait.

Trunk and pelvis kinematic analysis observed during the static trials revealed additional reliability concerns. Pelvis and trunk rotation angles showed low within-session reliability during session 2 (static 2 vs static 4), with ICCs of 0.43 and 0.50, respectively. Because trunk angles were calculated relative to the pelvis, variability in pelvic orientation likely contributed to the greater variability observed in trunk rotation measures. Fatigue, particularly in the residual limb hip flexors, may prompt compensatory strategies such as increased pelvic rotation toward the sound limb to maintain static balance and reduce muscular effort during standing. Discomfort or pain within the prosthetic socket during repeated walking trials can further contribute to asymmetrical weight distribution and postural adjustments, leading to pelvis and trunk rotation toward the sound limb during the final static trial.

Although SEM values for pelvis and trunk angles remained below 5° when comparing the 2 sessions, indicating good absolute reliability, ICC values were low (**Table 4**). This likely reflects variability in participants' static postures between sessions, which can affect ICC due to its sensitivity to between-subject variance.^14^ While ICC captures the proportion of variance due to true individual differences, inconsistent baseline positioning can reduce its values despite reliable within-subject measurements. In contrast, SEM reflects the within-subject consistency of repeated measurements and is unaffected by between-subject variability.^13^ Thus, the low ICCs likely result from postural variability rather than poor reliability.

It is also important to consider that the 5° threshold used to interpret SEM values is often applied in studies involving lower extremity joint angles which typically exhibit a much larger range of motion than the pelvis or trunk.^10^ Therefore, in these regions, even small absolute errors can be proportionally significant. Despite this, the consistently low SEM values observed still indicate good within-subject reliability and interpreting SEM alongside ICC offers a more complete assessment of measurement reliability.

Although each participant completed repeated sessions at approximately the same time of day to minimize within-subject variability due to prosthetic discomfort or fatigue, session times were not standardized across participants, which may have contributed to between-subject variability in marker placement and segment orientation. Static 1 was used as the baseline measurement for marker placement; however, it is possible that marker positioning in this trial was not fully representative of the participant's true anatomical alignment. Using an average of multiple static trials (e.g. static 1–3) might have provided a more stable baseline, potentially reducing variability in subsequent measures.

## CONCLUSION

Overall, findings support the reliability of pelvis marker placement and posture within a session in LLA populations but highlight challenges for between-session consistency, relevant for longitudinal studies. To improve consistency, we recommend collecting multiple trials per session, documenting prosthetic configuration, and standardizing participant posture using photographs or guides. Static trials remain useful for estimating segment lengths but may reflect habitual or compensatory postures rather than neutral alignment. Researchers should account for this when interpreting static kinematics. Ultimately, reliable gait analysis in LLA populations requires careful marker protocols, posture control, and awareness of static calibration limitations across timepoints.

## Figures and Tables

**Appendix 1:**
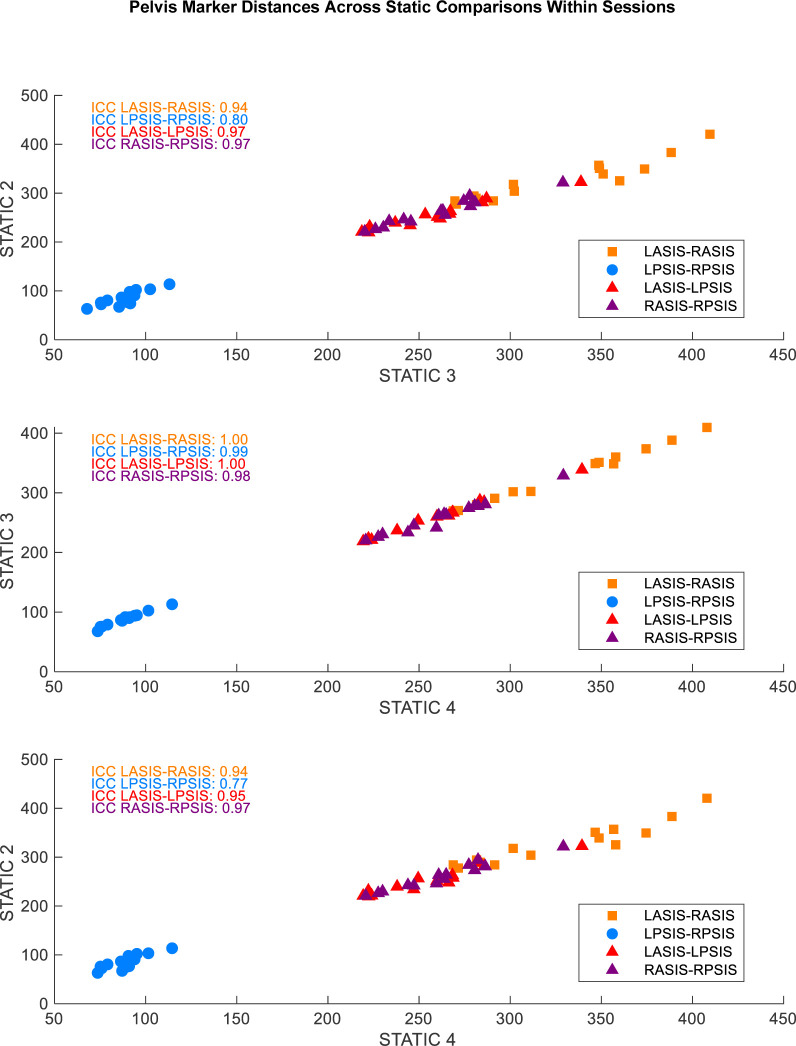
Individual pelvis marker distances across sequential static posture comparisons within sessions. Each subplot displays LASIS-RASIS, LPSIS-RPSIS, LASIS-LPSIS, RASIS-RPSIS with corresponding intraclass correlation coefficients (ICC) to indicate measurement reliability.

**Appendix 2:**
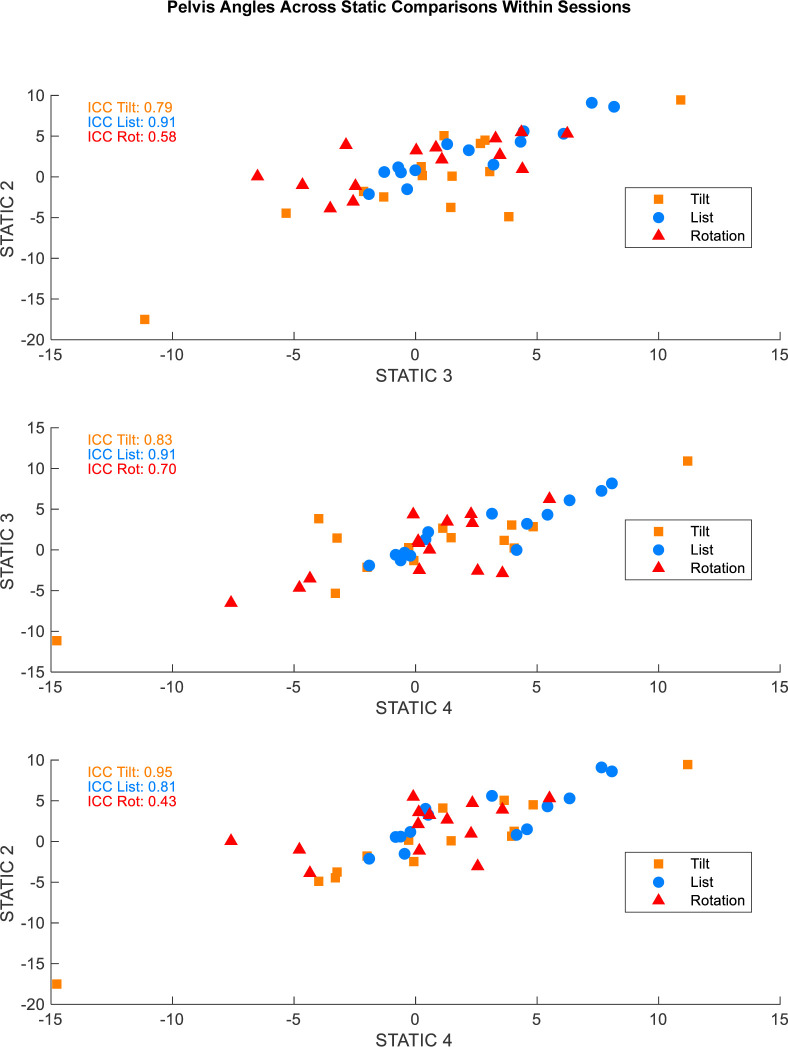
Individual pelvis angles across sequential static posture comparisons within sessions. Each subplot displays pelvis tilt, list, and rotation with corresponding intraclass correlation coefficients (ICC) to indicate measurement reliability.

**Appendix 3:**
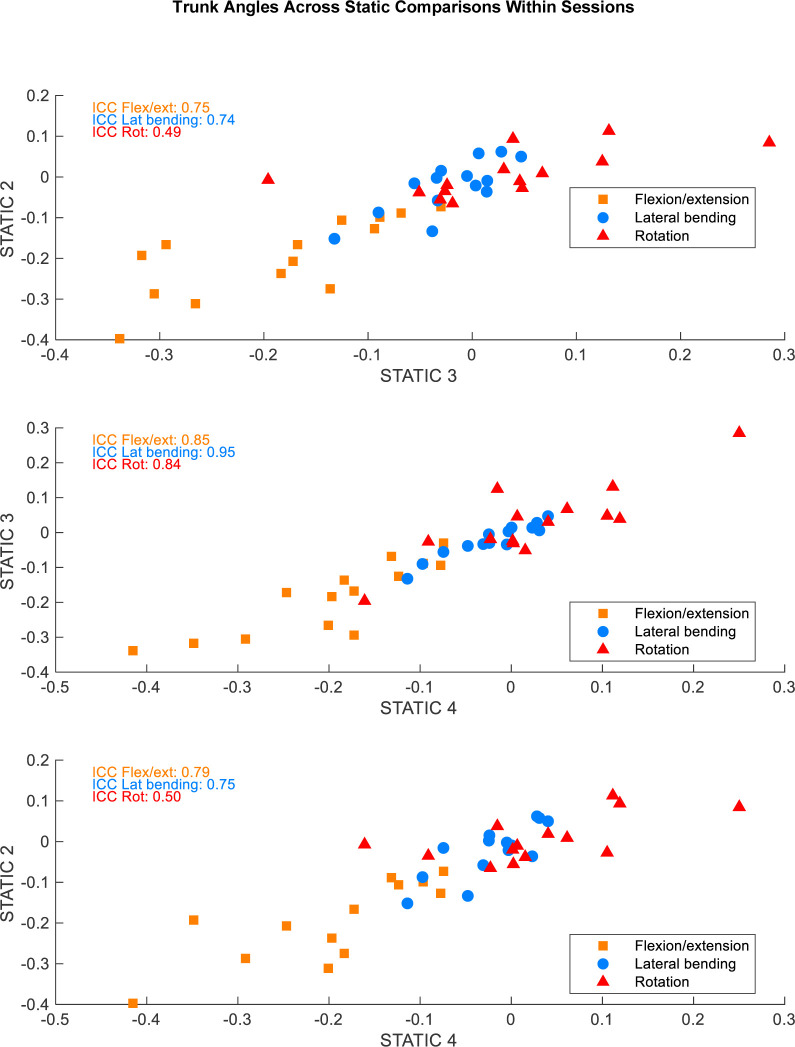
Individual trunk angles across sequential static posture comparisons within sessions. Each subplot displays trunk flexion/extension, lateral bending, and rotation with corresponding intraclass correlation coefficients (ICC) to indicate measurement reliability.

**Appendix 4:**
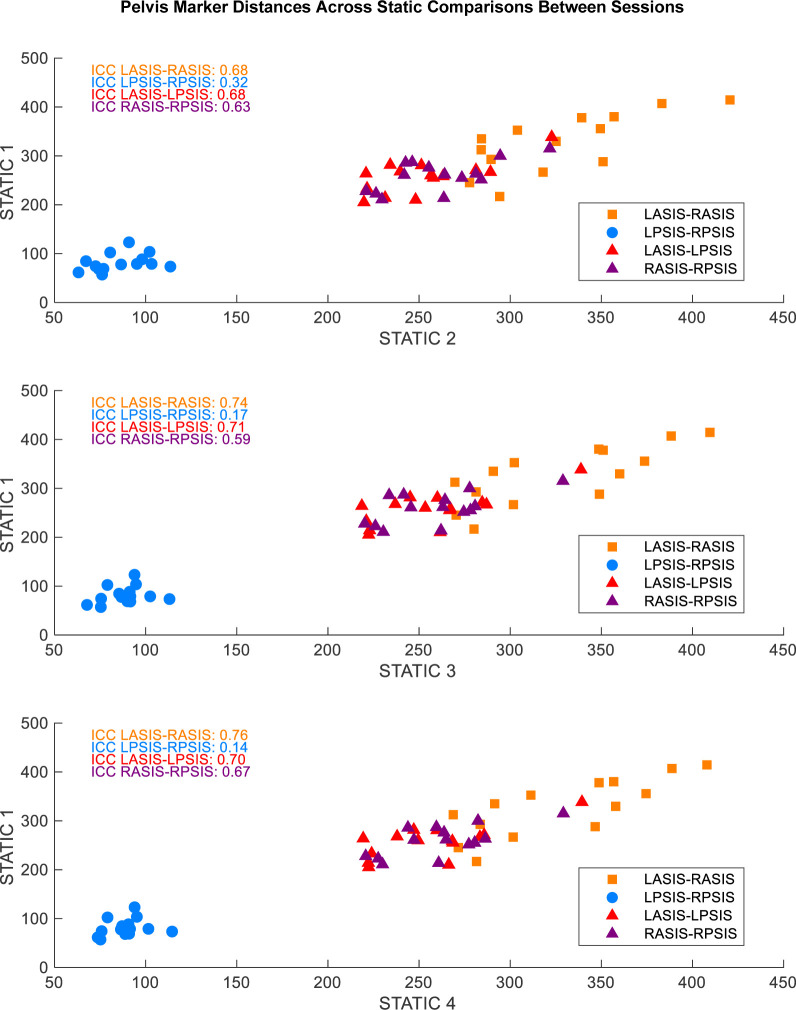
Individual pelvis marker distances across sequential static posture comparisons between sessions. Each subplot displays LASIS-RASIS, LPSIS-RPSIS, LASIS-LPSIS, RASIS-RPSIS with corresponding intraclass correlation coefficients (ICC) to indicate measurement reliability.

**Appendix 5:**
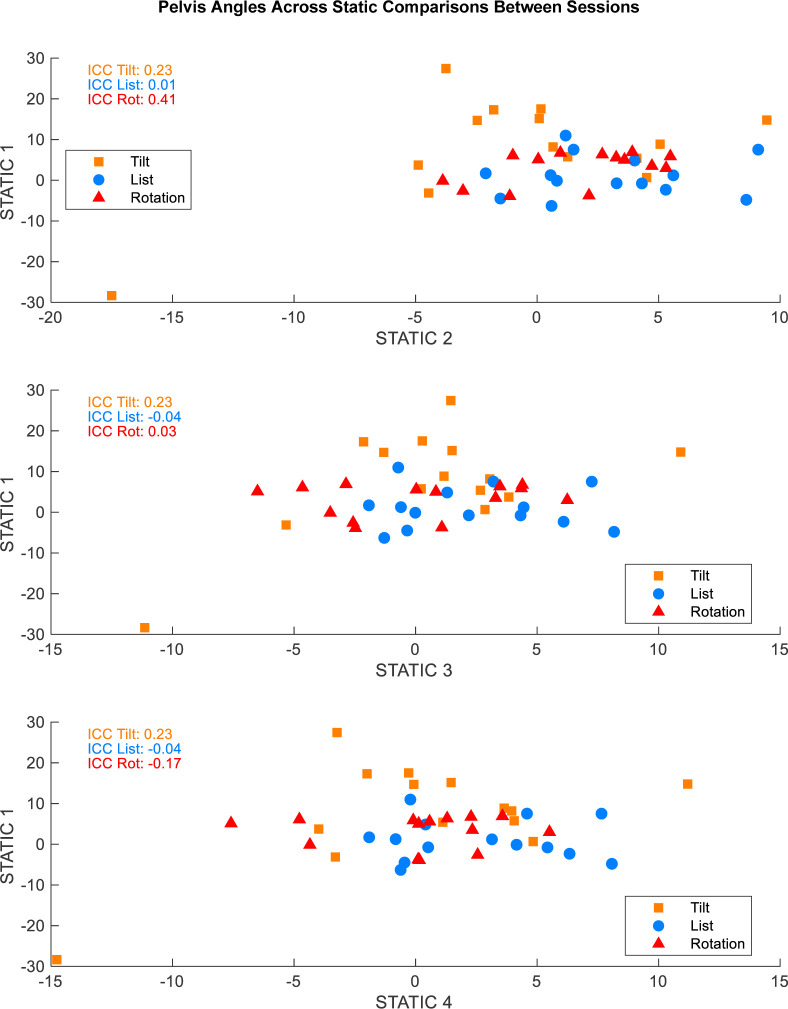
Individual pelvis angles across sequential static posture comparisons between sessions. Each subplot displays pelvis tilt, list, and rotation with corresponding intraclass correlation coefficients (ICC) to indicate measurement reliability.

**Appendix 6:**
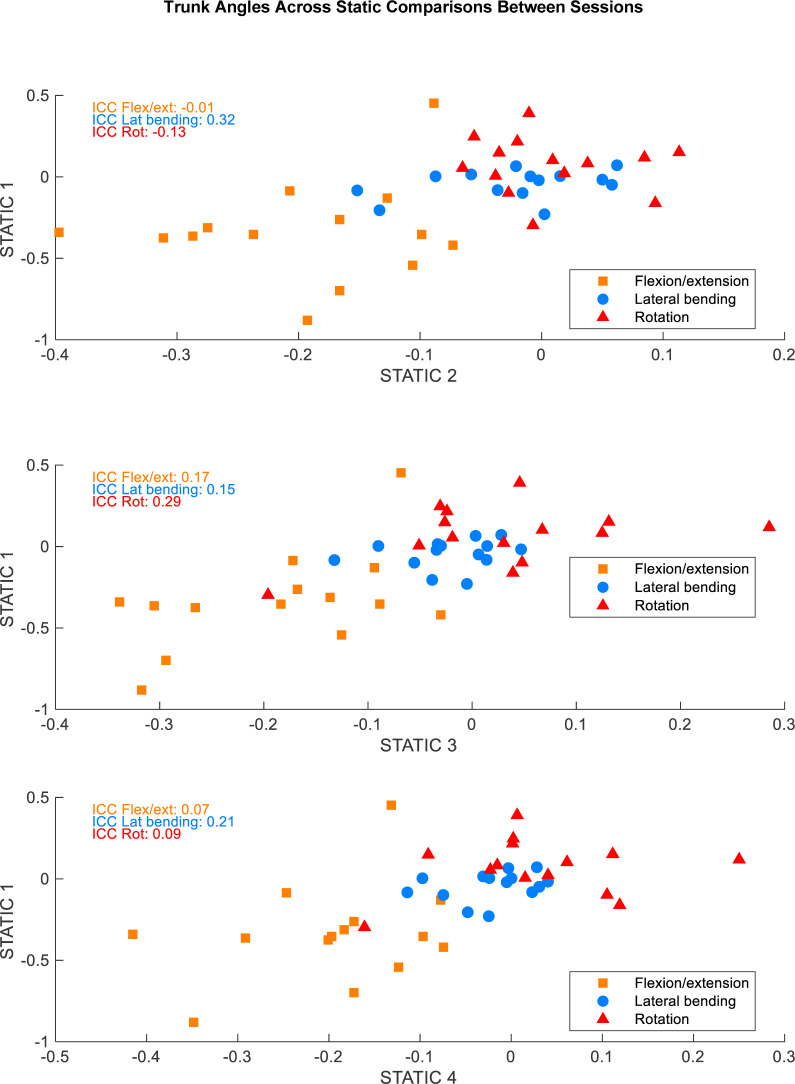
Individual trunk angles across sequential static posture comparisons between sessions. Each subplot displays trunk flexion/extension, lateral bending, and rotation with corresponding intraclass correlation coefficients (ICC) to indicate measurement reliability.
